# Inflammation and Oxidative-Stress Pathways Are Associated with Idiopathic Sudden Hearing Loss: A Genome-Wide Association Study in 15,494 Japanese Individuals

**DOI:** 10.3390/ijms27041836

**Published:** 2026-02-14

**Authors:** Ryosuke Kitoh, Shin-Ya Nishio, Yutaka Takumi, Shin-ichi Usami

**Affiliations:** 1 Department of Otorhinolaryngology-Head and Neck Surgery, Shinshu University School of Medicine, 3-1-1 Asahi, Matsumoto 390-8621, Nagano, Japan; takumi@shinshu-u.ac.jp; 2 Department of Hearing Implant Sciences, Shinshu University School of Medicine, 3-1-1 Asahi, Matsumoto 390-8621, Nagano, Japan; nishio@shinshu-u.ac.jp

**Keywords:** idiopathic sudden sensorineural hearing loss, genome-wide association study, conditional analysis, gene-based analysis, pathway enrichment, inflammation, oxidative stress

## Abstract

The etiology of idiopathic sudden sensorineural hearing loss (iSSNHL) remains unclear, and genome-wide genetic evidence is limited. We conducted a multicenter Japanese case–control genome-wide association study including 192 clinically defined iSSNHL cases and 15,302 controls aged ≥80 years without a history of hearing loss. After cross-platform SNP harmonization and imputation (Eagle/Minimac4), association testing was performed using dosage-based logistic regression in PLINK 2.0, adjusting for sex and principal components (PC1–PC10). Gene- and pathway-level analyses were conducted using MAGMA and the PANTHER overrepresentation test. Genomic inflation was modest (λ_GC = 1.04). Eight loci reached genome-wide significance (*p* < 5 × 10^−8^), led by *FHIT*, with additional loci near *LHX2*, *TRMT1L*, *MEGF10*, *SPATS1*, *SAMD5*, *MYT1L*, and *ID4*; 21 loci met the suggestive threshold (*p* < 1 × 10^−6^). MAGMA identified eight genes at FDR < 0.05 (*FHIT*, *TRMT1L*, *MEGF10*, *RNF2*, *SWT1*, *VAMP1*, *TAPBPL*, and *C9orf3*). These findings suggest that immune-inflammatory and cellular stress–homeostasis mechanisms may contribute to iSSNHL susceptibility and provide candidate loci for future replication and functional studies.

## 1. Introduction

Idiopathic sudden sensorineural hearing loss (iSSNHL) is a rapidly developing sensorineural hearing loss that occurs within 72 h. International epidemiological studies estimate an annual incidence of 5–27 per 100,000 people [1], whereas a nationwide Japanese epidemiological survey reported an incidence of 60.9 (95% CI 57.6–64.2) per 100,000 people [2]. Although local circulatory disturbance, viral infection, autoimmunity, and oxidative stress have been proposed as etiologies, the definitive mechanism remains unclear [3,4,5].

With respect to genetic contributions, previous studies have mainly employed candidate-gene association approaches. A recent systematic review summarizing 47 studies (5230 patients) and 68 genes reported associations with single nucleotide polymorphisms (SNPs) in several pathways related to coagulation/thrombosis (e.g., *F2*, *F5*, *MTHFR*, *ITGA2*/*ITGB3*), inflammation and immunity (e.g., IL-1, IL-6, *TNF*, *ICAM1*), oxidative stress and free-radical processing (e.g., *SOD1*, *GSTP1*, *GPX*, *GSR*, *NOS3*), as well as genes located in or near the HLA region [6].

Consistent with these observations, we previously conducted a two-stage candidate-gene association study using the Intractable Inner Ear Disease Gene Bank and found that *SOD1* rs4998557, together with *GSTP1* and *PRKCH* SNPs, may contribute to susceptibility to SSNHL, particularly among patients with hearing loss ≥60 dB and tinnitus [7]. Using the same cohort, we further demonstrated that SNPs in oxidative-stress and vascular genes (*GSR* rs2251780/rs3779647 and *NOS3* rs1799983) and in the glucocorticoid receptor gene *NR3C1* rs4912910 were associated with differences in hearing recovery after standardized corticosteroid therapy, suggesting that germline variations may influence not only disease risk but also prognosis and treatment response [8]. Nevertheless, most candidate-gene association studies, including our own, have been based on relatively small case–control series and have evaluated a limited number of loci, and the overall reproducibility of individual associations has been modest because of insufficient statistical power, multiple testing, population differences, and patient-recruitment bias [6].

As iSSNHL is likely a polygenic disorder to which many small-effect SNPs contribute, a hypothesis-free genome-wide association study (GWAS) followed by gene-level analyses (e.g., MAGMA) and pathway-level interpretation (pathway enrichment) is a more suitable approach than candidate-gene-based analysis. Indeed, while large-scale GWASs have been performed for age-related hearing loss and self-reported “hearing difficulty”, rigorously defined iSSNHL has rarely been subjected to a large GWAS because of its lower incidence [6].

In this study, we conducted a GWAS of iSSNHL using a multicenter Japanese cohort. By connecting single-nucleotide polymorphism (SNP)-level results with gene- and biological pathway-level analyses, we aimed to provide genome-wide evidence and generate testable hypotheses regarding the molecular mechanisms underlying disease susceptibility.

## 2. Results

### 2.1. Participant Characteristics

This study analyzed an iSSNHL patient cohort comprising 192 samples from 11 facilities. Age at onset was available for 181 cases and unavailable for 11 cases: the mean age at onset for the 181 cases was 56.1 ± 15.4 years (range, 9–86 years; median, 59 years (interquartile range [IQR] 46–67)). The cohort included 109 males and 83 females. The mean hearing level (average of 250, 500, 1000, 2000, and 4000 Hz) in the affected ear before treatment was as follows: <40 dB in 19 cases, ≥40 dB and <60 dB in 41 cases, ≥60 dB and <90 dB in 78 cases, and ≥90 dB in 54 cases.

The final dataset used for association testing comprised 15,494 individuals (192 cases and 15,302 controls), selected under the criteria described in the Methods.

### 2.2. GWAS Results (SNP-Level Analysis)

**Figure 1:**
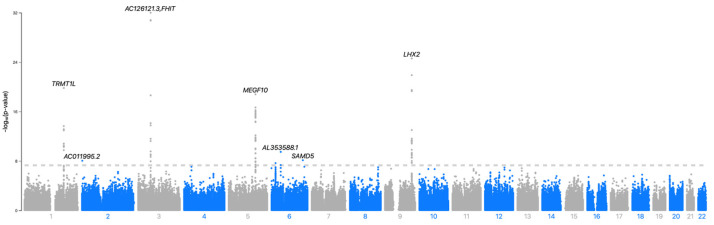
Genome-wide Manhattan plot (build = GRCh37/hg19). The y-axis shows −log_10_P and the x-axis the genomic position. Points are colored by chromosome (alternating colors for adjacent chromosomes). The dashed line indicates the genome-wide significance threshold (*p* = 5 × 10^−8^). The top loci (*FHIT*, *LHX2*, *TRMT1L*, *MEGF10*, *SPATS1*, *SAMD5*, *MYT1L*, *ID4*) are annotated.

**Figure 2:**
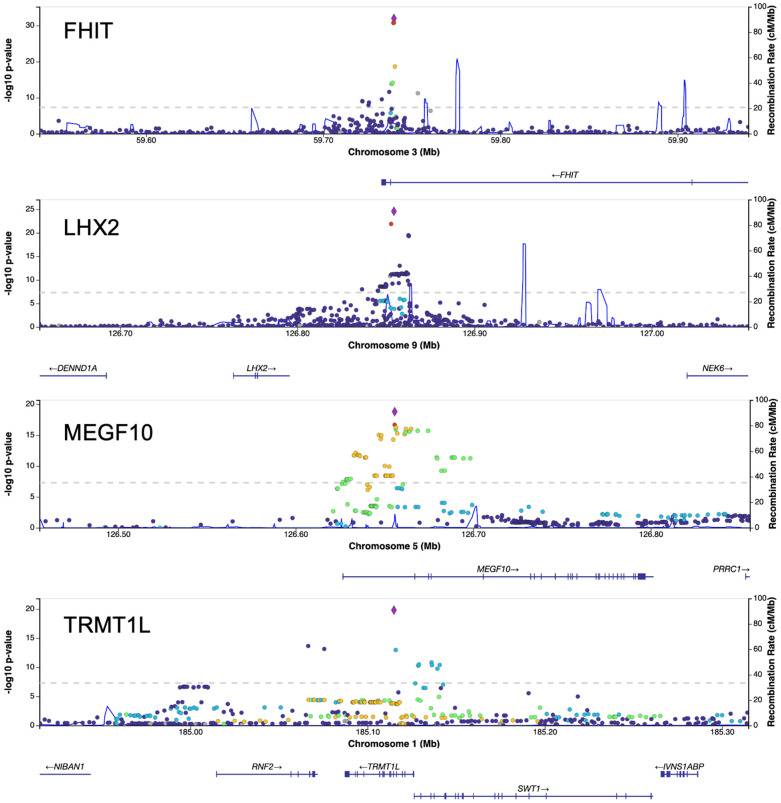
Regional association plots for the four top loci (LocusZoom web portal (https://locuszoom.org/)). Panels were generated with LocusZoom (web portal built on LocusZoom.js) using the 1000 Genomes Phase 3 EAS LD and GRCh37/hg19 coordinates; window size ±200 kb around the lead variant. The purple diamond marks the lead variant; dot colors indicate LD r^2^ with the lead. The blue curve represents the recombination rate (cM/Mb). The dashed line denotes *p* = 5 × 10^−8^ (−log_10_P = 7.30). Lower tracks display GWAS Catalog hits and gene models with transcriptional direction. Peaks are centered on rs6803403 (*FHIT*), rs72759216 (*LHX2*), rs246887 (*MEGF10*), and rs6684586 (*TRMT1L*).

Genomic inflation was modest (λ_GC = 1.04), indicating no major systematic bias. We identified eight loci that reached genome-wide significance (*p* < 5 × 10^−8^) (see Table 1). The rsIDs in Table 1 were unified according to dbSNP (version 155; GRCh37). Only variants without an assigned rsID are shown as “chr:pos (GRCh37)”. Figure 1 shows the genome-wide Manhattan plot, and Figure 2 shows LocusZoom regional association plots for the four top loci: rs6803403 (*FHIT*, chromosome 3), rs72759216 (*LHX2*, chromosome 9), rs246887 (*MEGF10*, chromosome 5), and rs6684586 (*TRMT1L*, chromosome 1). In addition to the eight genome-wide significant loci, we identified 21 loci that fulfilled the suggestive threshold (5 × 10^−8^ ≤ *p* < 1 × 10^−6^), as summarized in Table 2.

In each region, the most significant variant (purple diamond) and a cluster of additional variants with high LD also showed strong association (dashed line at *p* = 5 × 10^−8^), providing the input for the subsequent COJO analyses. The genomic context in the plots is consistent with the annotation in Table 1 (*FHIT*/*TRMT1L*/*MEGF10*: overlap; *LHX2*: intergenic). For each lead variant, the complete list of genes within ±250 kb together with the overlap/distance labels defined in Methods is provided in Supplementary Table S1. To facilitate interpretation of the effect sizes, dosage-based allele frequencies and dosage-derived minor allele counts for each lead SNP are provided in Supplementary Table S2.

### 2.3. Conditional and Joint Analysis Results

Using GCTA-COJO (stepwise selection; COJO-SLCT), we identified multiple independent signals at *FHIT* (three signals), *TRMT1L* (three signals), and *LHX2* (two signals) (Table 3). These remained clearly detectable in excl models; i.e., when conditioning was performed on the other COJO-selected leads while excluding the target lead, supporting their independence. In contrast, *MEGF10*, *ID4*, and *SPATS1* each harbored a single lead, and the post-all minimum pC did not reach genome-wide significance at these loci (Table 3). *SAMD5*/*MYT1L* did not yield independent signals in the selection step (No. selected leads = 0). For *TRMT1L*, the post-all minimum pC was 5.13 × 10^−8^, close to the genome-wide significance threshold. Given the approximate nature of external LD references, we refrained from over-interpretation.

### 2.4. Gene-Level Results (MAGMA)

Gene-based testing with MAGMA (16,025 genes) identified eight genes that reached statistical significance after BH–FDR correction (FDR < 0.05; Table 4). *FHIT* showed the strongest association (*p* = 2.3 × 10^−30^, FDR = 3.6 × 10^−26^), followed by *TRMT1L* and *MEGF10* (*p* ≈ 7.2–7.7 × 10^−16^). *RNF2*, *SWT1*, *VAMP1*, *TAPBPL* and *C9orf3* also remained significant after correction. Notably, *FHIT*, *TRMT1L* and *MEGF10* overlapped the top SNP-level loci, whereas *RNF2*, *SWT1*, *VAMP1*, *TAPBPL* and *C9orf3* emerged only at the gene level.

### 2.5. Pathway Enrichment Results (PANTHER)

Based on the GWAS results, we constructed a GWAS-derived gene set for pathway analysis. For loci that reached genome-wide significance, genes located within a ±250-kb window around each lead SNP were taken from the positional gene lists in Supplementary Table S1 and cleaned according to the procedure in Methods (Section 4.9), leaving 28 genes. For loci that met the suggestive threshold in Table 2, we selected the nearest gene(s) at each independent locus and, after the same cleaning, obtained 16 genes summarized in Supplementary Table S3. Together, these 28 and 16 genes formed a 44-gene GWAS-derived “clean set,” which we analyzed with the PANTHER Overrepresentation Test (PANTHER v19.0) using the *Homo sapiens* genome as the reference list and Fisher’s exact test with Benjamini–Hochberg FDR correction, with UNCLASSIFIED categories excluded.

No pathway reached the predefined significance threshold of FDR < 0.05. Nevertheless, several pathways showed nominal enrichment when ranked by fold enrichment and raw *p* value (Table 5). Among these, the gonadotropin-releasing hormone receptor pathway and the “inflammation mediated by chemokine and cytokine signaling” pathway were notable. The latter pathway included *IFNG*, *NFKBIE*, and *ITPR2*, which is consistent with a potential contribution of immune-inflammatory mechanisms to iSSNHL susceptibility. As no pathway passed FDR < 0.05, these pathway-level observations should be interpreted as exploratory (hypothesis-generating).

## 3. Discussion

### 3.1. Comparison with Previous Studies and the Originality of This Work

Early genetic studies of iSSNHL employed candidate-gene association study-based approaches, focusing on specific genes such as HLA types, inflammatory cytokines (e.g., IL6 and *TNF*), coagulation-related genes (*F2*, *F5*, and *MTHFR*), and oxidative stress-related genes (e.g., *SOD1*, *GSTP1*, *GPX1*/*3*, *GSR*, and *PON1*), as summarized in a recent systematic review [6]. The reproducibility of these findings has been hindered by a combination of small sample sizes, the burden of multiple comparisons, and population heterogeneity [6]. In contrast, GWASs of hearing-related traits that focused on age-related hearing loss (ARHL), self-reported “hearing difficulty,” tinnitus, and vertigo have been performed previously [9,10,11,12]. More recently, a Mendelian randomization analysis using FinnGen iSSNHL cases explored the causal relationship with inflammation-related SNPs [13]. Nevertheless, case–control GWASs of clinically well-phenotyped iSSNHL, diagnosed under unified criteria and accompanied by detailed clinical information, remain scarce.

The originality of the present study can be summarized as follows:

Definition of phenotype and multi-institutional accrual: iSSNHL samples were collected through the Intractable Inner Ear Diseases Gene Bank Japan, which involves 23 collaborating institutions across Japan under uniform diagnostic criteria; the present GWAS analyzed 192 cases accrued from 11 institutions (including Shinshu University).

Control design: We adopted a “resilient” control phenotype, which was defined as individuals aged ≥80 years at sampling without a history of hearing loss from BBJ, to minimize the risk of future incident iSSNHL cases.

Multilayer interpretation: SNP-level GWAS identified eight genome-wide significant loci (*FHIT*, *LHX2*, *TRMT1L*, *MEGF10*, *SPATS1*, *SAMD5*, *MYT1L*, and *ID4*), and gene-based analysis (MAGMA) identified eight genes at FDR < 0.05 (*FHIT*, *TRMT1L*, *MEGF10*, *RNF2*, *SWT1*, *VAMP1*, *TAPBPL*, and *C9orf3*). The PANTHER pathway overrepresentation analysis did not yield any pathway passing FDR < 0.05; therefore, pathway-level observations based on nominal *p* values should be considered exploratory and require replication.

Taken together, this work represents an early data-driven GWAS of clinically well-phenotyped iSSNHL in a Japanese cohort and provides a framework for future replication and functional follow-up. In a cohort of 192 cases and 15,302 controls, we observed SNP-level and gene-level signals consistent with immune-inflammatory and cellular stress–homeostasis mechanisms; however, pathway-level signals from PANTHER were not FDR-significant and are therefore hypothesis-generating. In the SNP-level analysis, rs72759216 near the *LHX2* locus was among the strongest associations in our GWAS, even though *LHX2* itself did not reach gene-based significance in MAGMA and did not anchor an FDR-significant pathway. *LHX2* encodes a LIM-homeodomain transcription factor essential for forebrain development and thalamocortical axonal guidance, including pathways from the auditory thalamus [14]. Its implication in an adult-onset disorder such as iSSNHL is therefore intriguing but should be interpreted cautiously; we consider this a developmental susceptibility hypothesis that will require replication and targeted functional studies to clarify the causal gene and mechanism.

### 3.2. Two Pathways of the Pathophysiological Model

#### 3.2.1. The Inflammation/Immunity Pathway: Integration of *IFNG* and the NF-κB Pathway

Integrating the SNP-level GWAS results with MAGMA and PANTHER suggests an inflammation/immunity axis centered on the *IFNG*, *ITPR2*, and *NFKBIE* loci. In our data, lead variants near *IFNG* and *ITPR2* reached the suggestive threshold (*p* ≤ 1 × 10^−6^), whereas *NFKBIE* lay within the ±250 kb window of one of the genome-wide significant loci; these genes were included in the GWAS-derived gene set and mapped to the “inflammation mediated by chemokine and cytokine signaling” pathway in PANTHER. However, because no pathway passed FDR < 0.05, this pathway-level interpretation should be considered hypothesis-generating and requires replication.

(1) IFN-γ/Th1 pathway.

In autoimmune sensorineural hearing loss (ASNHL), T cells that react to inner ear antigens produce IFN-γ. This suggests that an acute/subacute Th1-skewed environment can develop in sensorineural hearing loss [15]. In inner ear models, IFN-γ pretreatment activates JAK1/2–STAT1 and increases susceptibility to TNFα toxicity via TNFR1-dependent caspase1. Moreover, IFN-γ and TNFα act synergistically to aggravate cisplatin-induced hair cell injury [16]. Spiral ligament fibrocytes can produce TNFα in response to inflammatory stimuli, suggesting that local cellular constituents may initiate inflammation. The genetic observations near *IFNG* in our GWAS are compatible with such upstream sensitization mechanisms.

(2) Ca^2+^–NF-κB response.

*ITPR2* mediates intracellular Ca^2+^ release and is involved in signal amplification under inflammatory stimulation [17]. *NFKBIE* restrains NF-κB activation and variations in this brake may shift stress response thresholds. Temporal bone pathology suggests that classical vascular occlusion or membrane rupture alone cannot explain all iSSNHL cases, raising the possibility that pathological activation of cellular stress pathways (including NF-κB) contributes to disease [3]. In mice, NF-κB deficiency is associated with auditory nerve degeneration and exacerbated noise-induced hearing loss [18], and reports variably position NF-κB as both protective and detrimental within cochlear oxidative-stress responses.

(3) Integration and therapeutic implications.

Dexamethasone-mediated oto-protection has been linked to PI3K/Akt/NF-κB signaling [19]; thus, genetic heterogeneity along the NF-κB pathway could underlie differential steroid responsiveness in iSSNHL. In summary, our findings related to the inflammatory pathway suggest the existence of genetically determined thresholds within a cascade comprising IFN-γ-driven sensitization, Ca^2+^ dynamics via *ITPR2*-mediated amplification, and NF-κB responses modulated by *NFKBIE*. Prospective stratification combining IFN-γ/TNFα profiles and genotype may help to identify individual inflammatory drivers and response pathways.

#### 3.2.2. The Non-Inflammatory Pathway: Oxidative Stress and Cellular Homeostasis (FHIT/TRMT1L/MEGF10)

Although our focus is on inflammation and immunity, the top signals displaying concordance between GWAS and MAGMA (*FHIT*, *TRMT1L*, and *MEGF10*) support a complementary pathway of cell-intrinsic stress responses and homeostatic maintenance that may operate upstream or downstream of inflammation.

*FHIT* spans the common fragile site FRA3B and contributes to genome stability by coping with replicative stress and the DNA damage checkpoint/repair process [20]. Loss of *FHIT* increases replication stress and double-strand breaks, thereby promoting genome instability via dysregulated stress responses [21]. *FHIT* also cooperates with *FDXR* (ferredoxin reductase) in mitochondria to regulate apoptosis thresholds under severe oxidative stress [22]. In the inner ear, ischemia/reperfusion and noise exposure elevate lipid peroxidation and increase 8-oxo-7,8-dihydro-2’-deoxyguanosine (8-oxo-dG), among other markers; thus, the known roles of *FHIT* in genome stability and modulation of oxidative stress may contribute to interindividual susceptibility to acute threshold shifts [23,24].

*TRMT1L* participates in tRNA methylation and post-transcriptional modification, and is thereby linked to protein quality control and stress granule dynamics under oxidative stress [25,26]. Similar to *FHIT*, SNPs in *TRMT1L* could influence the handling of oxidative stress relevant to iSSNHL onset.

*MEGF10* is a receptor that recognizes and engulfs apoptotic cells and cell debris (efferocytosis), acting in concert with opsonins such as C1q and phagocytic “eat me” signals [27]. In the central nervous system, *MEGF10* binds C1q to mediate clearance of apoptotic cells by astrocytes [28]. In the mouse cochlea (from P8 to P20), *MEGF10* shows relatively higher expression in supporting cells in sc/snRNA-seq datasets (visualized in gEAR) [29]. During hair cell injury in the inner ear, the clearance of dead hair cells/fragments by supporting cells is thought to curb secondary inflammation and help maintain ionic homeostasis [30]. While indirect, these mechanisms suggest that differences in *MEGF10* function could modulate iSSNHL susceptibility.

### 3.3. Clinical Implications

Our GWAS indicates genetic signals spanning inflammation and oxidative-stress responses, which is consistent with the conventional theory that the disease is multifactorial rather than being caused by a single mechanism. Clinically, stratification and treatment choices should consider two pathways: (a) inflammation, and (b) oxidative stress/cellular homeostasis. Recent reviews and clinical studies support the roles of inflammation, metabolic dysregulation, and oxidative stress, and suggest the utility of blood-based markers for prognosis [31,32].

Potential applications include: Stratification by “visualizing” the inflammation pathway in combination with genotype. At initial presentation, low-cost NLR/PLR indices derived from routine blood counts are accumulating evidence of an association with onset and prognosis [33]. IFN-γ and TNFα levels can rise acutely and decline with recovery [34]. Combining such acute phase biomarkers with genotypes (e.g., *IFNG*/*NFKBIE*) may enable future risk stratification and therapy selection.

NF-κB functional readouts and oxidative-stress markers. NF-κB is a hub of cochlear inflammatory responses and is activated by noise and other stressors [34,35]. In research settings, peripheral blood mononuclear cell (PBMC) assays involving short-term stimulation followed by p65 (RelA) phosphorylation and NF-κB target gene expression can serve as functional readouts [34]. As steroids classically suppress NF-κB activity [35], pairing NF-κB-related genotypes with PBMC readouts to examine differences in steroid responsiveness is feasible and merits evaluation in prospective studies. Oxidative-stress markers such as 8-OhdG and lipid peroxidation (MDA/TBARS) change in SSNHL and are emphasized in pathophysiological reviews [5,32]. Exploratory use of these markers alongside genotypes (e.g., *FHIT*/*TRMT1L*) could support stratification by stress tolerance.

### 3.4. Strengths and Limitations

Although the case series is moderate in size for GWAS, we leveraged a large control set, achieving a favorable λ_GC = 1.04. However, in a sensitivity analysis requiring Rsq > 0.8, genome-wide significance persisted for *FHIT*, *TRMT1L*, *MEGF10*, and *LHX2*, while other variants were not significant, and replication is therefore necessary for these. Furthermore, the genes (variants) reaching significance here require functional validation (expression, eQTL, cellular/animal models) to confirm causality. Several lead variants displayed very large ORs with wide confidence intervals, consistent with low allele counts for low-frequency variants (Supplementary Table S2); these estimates should be interpreted cautiously and require replication.

An additional limitation is the extreme age imbalance between cases (predominantly midlife onset) and controls (≥80 years), which is inherent to our “resilient control” design. Although germline genotypes are fixed and do not change with age, using very old controls may introduce residual confounding via age-related survival or birth-cohort effects that can influence allele frequencies. We did not perform age-matched subsampling or include age as a covariate, as age was an explicit component of the control definition; replication in age-matched cohorts and alternative designs will be important.

In addition, our analyses were limited to germline genetic variation and did not assess epigenetic regulation (e.g., DNA methylation and chromatin-level regulatory mechanisms) or gene-environment interactions that may influence iSSNHL susceptibility. Future studies integrating epigenomic and transcriptomic profiling with GWAS will be important to clarify the regulatory mechanisms underlying the implicated inflammatory and oxidative-stress pathways.

## 4. Materials and Methods

### 4.1. Study Design and Ethics

This study was a multicenter case–control genome-wide association study. The protocol was approved by the Institutional Review Board of Shinshu University School of Medicine (Approval No. 5528; date of approval: 31 May 2022), and written informed consent was obtained from all participants.

### 4.2. Cases

Genomic DNA was obtained from peripheral blood samples of patients with iSSNHL who were registered in the Intractable Inner Ear Diseases Gene Bank Japan. This biobank includes samples collected at 23 collaborating research facilities across Japan, as reported previously [7], and contains detailed clinical information, including sex and age at onset, hearing-loss severity, accompanying symptoms (e.g., tinnitus and vertigo), treatment, and outcomes. Of these, 11 facilities (including Shinshu University) contributed the 192 case samples analyzed in the present GWAS.

The diagnostic criteria for SSNHL unified the domestic standards, based on the 2018 edition of the Clinical Practice Guidelines for the Diagnosis and Management of Acute Sensorineural Hearing Loss (Japan Audiological Society; Kanehara & Co., Tokyo, Japan; in Japanese), as well as the revised criteria proposed by the Research Group on Acute Severe Sensorineural Hearing Loss in 2012 [36]. The summary of the specific diagnostic criteria is as follows:

Main symptoms: Sudden onset of sensorineural hearing loss, which is usually severe and of unknown etiology.

For reference: Hearing loss of ≥30 dB over three consecutive frequencies, with the possibility of progressive deterioration within 72 h, is also specified in the guidelines.

### 4.3. Controls

In case–control studies of iSSNHL, individuals without a prior diagnosis at the time of DNA sampling may still develop iSSNHL later in life. To reduce this potential misclassification, we defined controls as individuals aged ≥80 years at DNA sampling with no history of hearing loss. This design was supported by our nationwide epidemiological survey of >3400 individuals with iSSNHL, in which most cases occurred before age 80 and only 3.1% had onset at age ≥80 years [36]. Accordingly, age was treated as part of the control phenotype definition rather than a covariate in the primary association model.

To obtain a sufficiently large control set meeting these criteria, we used the Biobank Cross Search system to identify eligible samples across Japanese biobanks. BioBank Japan (BBJ) provided the majority of eligible samples. Genotype data for 15,494 BBJ participants who met the above criteria and were enrolled in 47 common disease projects [37] were obtained from the National Bioscience Database Center (NBDC), Japan Science and Technology Agency, via the Japanese Genotype-phenotype Archive (JGA; dataset ID: JGAD000123) after ethical approval and under Data Access Committee-approved data-use agreements (Application ID: JDU000716002) [38]. Following the sample-level QC procedures described below (including missingness filtering, relatedness filtering, and population-structure assessment), 15,302 participants remained and were used as controls in the final GWAS.

### 4.4. Genotyping, Data Harmonization, Imputation, and Quality Control

#### 4.4.1. Genotyping Arrays

Cases were genotyped using Affymetrix Axiom Japonica Array^®^ v2 (Thermo Fisher Scientific, Waltham, MA, USA) at Toshiba, Co., Ltd. (Tokyo, Japan) according to the manufacturer’s protocol/instructions. Data processing followed StaGen’s (StaGen, Tokyo, Japan) in-house standard operating scripts, in the order below.

#### 4.4.2. Format Conversion and Initial Alignment

The BBJ final reports were converted to PLINK format (final report → tped/tfam → bed/bim/fam). Based on BLAST (2.17.0)-derived coordinates and allele information, all genotypes were unified to the forward strand.

#### 4.4.3. Within-Group QC

SNP-level filters were applied stepwise to cases and controls separately: missingness (option --geno 0.01), minor allele frequency (MAF) ≥ 0.01, and Hardy–Weinberg equilibrium (HWE) *p* ≥ 1 × 10^−4^ in controls. At the sample level, individuals with missing call rates > 2% were excluded, and outliers in heterozygosity (median ± 4 SD) were removed.

#### 4.4.4. Harmonization (Common SNP Definition)

To avoid inconsistencies between different arrays, a unified SNP identifier was generated: CHR: POS: Minor: Major. Only SNPs shared between cases and controls under this common definition were extracted and merged.

#### 4.4.5. VCF Conversion and Imputation

VCFs for each chromosome from the common SNP set were submitted to the local Michigan Imputation Server (https://imputationserver.sph.umich.edu/ (accessed on 3 January 2026)) for pre-phasing using Eagle v2.4 (https://data.broadinstitute.org/alkesgroup/Eagle/ (accessed on 3 January 2026)), followed by imputation using Minimac4 v4.1.6 (https://genome.sph.umich.edu/wiki/Minimac4 (accessed on 3 January 2026)) with the 1000 Genomes Project Phase 3 East Asian (EAS) reference and GRCh37/hg19 [39].

#### 4.4.6. Post-Imputation QC

From the imputed VCFs, variants with Rsq ≥ 0.3 and MAF > 0.001 were retained for dosage-based analyses, generating the final GWAS input. As the imputation outputs lacked rsIDs, we assigned these from the latest dbSNP chromosome-wise VCF for GRCh37/hg19 (dbSNP version 155 (GRCh37), Release 8 April 2021), as detailed below. Finally, the total number of variants entered into the GWAS (autosomes chr1–22) was 5,187,415.

#### 4.4.7. rsID Assignment

Variants were matched by CHR: POS: REF: ALT in order to assign current, valid dbSNP rsIDs. Ambiguous A/T and C/G alleles were checked to ensure there was no strand discordance with the GRCh37 reference strand. rsIDs were only assigned for multiallelic sites when the REF and ALT allele pair matched exactly. Variants that were not registered in dbSNP or could not be uniquely resolved were not assigned an rsID and were displayed as “chr:pos” (GRCh37).

#### 4.4.8. Display Rules

In the main text and tables, rsIDs are displayed preferentially (e.g., rs123456). Only variants without an assigned rsID are shown as “chr:pos”. For internal procedures such as COJO, the position ID (posID = “CHR:POS”) was used consistently; reader-facing materials include rsIDs in parallel (see Section 4.7 for COJO details).

### 4.5. Population Structure and Sample QC

From the set of common SNPs, individuals with missing call rates > 2% were excluded, as were heterozygosity outliers (median ± 4 SD). Cryptic relatedness was evaluated using identity-by-descent (IBD) estimates in PLINK, and related individuals were removed using a threshold of PI_HAT > 0.1875. After sample-level QC and population-structure assessment, the final analytic dataset comprised 192 cases and 15,302 controls (N = 15,494). After linkage disequilibrium (LD) pruning (MAF ≥ 0.05, HWE *p* ≥ 1 × 10^−4^, r^2^ < 0.1), principal component analysis (PCA) was performed using flashPCA v2.1 (https://github.com/gabraham/flashpca (accessed on 2 January 2026)) with outliers (mean ± 6 SD on each PC) excluded [40]. The final regression model included sex and PC1–PC10.

### 4.6. GWAS (SNP-Level Analysis)

Logistic regression analysis was performed using dosage (DS) from imputed VCFs (Rsq ≥ 0.3, MAF > 0.001) using PLINK v2.0 (https://www.cog-genomics.org/plink/2.0/ (accessed on 2 January 2026)) (generalized linear model module), with case–control status as the outcome, DS as the predictor, and sex plus PC1–PC10 as covariates [41,42]. Genome-wide significance was defined as *p* = 5 × 10^−8^, and suggestive significance as *p* = 1 × 10^−6^. The genomic inflation factor λ_GC was computed using autosomal SNPs with Rsq ≥ 0.8 and MAF > 0.01. If a lead SNP overlapped the gene coding sequence, the corresponding gene was annotated as an overlap; otherwise, the nearest gene was assigned based on the shortest distance to a gene-body boundary. Gene boundaries were taken from NCBI37.3.gene.loc (GRCh37/hg19). For positional context, we defined “nearby genes” as those whose gene bodies fall within ±250 kb of each lead variant. Regional association plots were generated with the LocusZoom web portal (built on LocusZoom.js) [43], with 1000 Genomes Phase 3 EAS LD and GRCh37/hg19 coordinates. Each panel displays −log_10_P against the genomic position within a ±200 kb window centered on the lead variant (purple diamond). Dots are color-coded by LD r^2^ with the lead, while gray indicates variants for which no LD information is available. The recombination rate (cM/Mb) is shown in blue. The dashed line denotes *p* = 5 × 10^−8^ (−log_10_P = 7.30). Age was not included as a covariate in the primary model because controls were deliberately defined as individuals aged ≥80 years (“resilient controls”) to minimize future case misclassification; potential limitations of this design are discussed in the Discussion (Section 3.4).

### 4.7. Conditional and Joint Analysis (COJO)

For genome-wide significant loci, we evaluated the presence of multiple independent signals using GCTA-COJO v1.94.1 (https://yanglab.westlake.edu.cn/software/gcta/ (accessed on 2 January 2026)). For each region (± 2 Mb around the index SNP), we performed conditional analyses using 1000 Genomes Phase 3 EAS as the linkage disequilibrium (LD) reference (*n* = 504; PLINK format). SNP IDs were unified as posID = “CHR:POS”, and only variants common to the jma file (summary statistics) and the LD reference were used.

First, stepwise selection of independent lead SNPs was conducted with GCTA-COJO (step-wise selection; COJO-SLCT) using the threshold *p* < 5 × 10^−8^, collinearity cutoff of 0.9, and a ± 2 Mb window. Next, each selected lead SNP was re-estimated conditioning on the other leads, and regional plots were generated for pre- (unadjusted) and post-excl statuses. Finally, post-all estimates were obtained with GCTA-COJO (conditional model including all selected leads), and the minimum *p*C within each region was summarized in a table. Visualization was performed using a minimal script to highlight the independent leads with large diamonds and only label the focal lead.

### 4.8. Gene-Level Analysis (MAGMA)

Gene-level associations were assessed using MAGMA v1.10 (https://ctg.cncr.nl/software/magma (accessed on 2 January 2026)) [44] in the summary-statistics mode. SNPs were mapped to genes using the NCBI37.3.gene.loc definitions (GRCh37/hg19) with a ±0 kb window around the gene body. The SNP-wise multi (“multi”, P_MULTI) gene test was applied, using the 1000 Genomes Project Phase 3 East Asian (EAS) panel as the LD reference. We set the sample size modifier to N = 15,494 (cases + controls). Statistical significance after multiple testing was defined using a Benjamini–Hochberg false discovery rate (BH-FDR) threshold of <0.05 across all autosomal genes tested (*n* = 16,025).

### 4.9. Pathway Enrichment Analysis (PANTHER)

We performed a pathway overrepresentation analysis of GWAS-derived loci using PANTHER (http://www.pantherdb.org/) [45]. First, we identified lead SNPs that met either genome-wide significance (*p* < 5 × 10^−8^) or the suggestive threshold (5 × 10^−8^ ≤ *p* < 1 × 10^−6^) in the SNP-level analysis. For genome-wide significant loci, “nearby genes” were defined as genes whose gene bodies fell within ±250 kb of each lead variant, based on NCBI build 37.3 gene coordinates (NCBI37.3.gene.loc; GRCh37/hg19).

For suggestive loci, we deliberately took a more conservative approach to avoid overinterpreting weaker association signals. Instead of including all genes within ±250 kb, we used the “Top loci” summary from the LocusZoom web portal (LocusZoom.js; 1000 Genomes Phase 3 East Asian [EAS] LD; GRCh37) to define independent loci and then selected only the nearest gene(s) reported for each lead SNP.

Gene symbols were harmonized against the GRCh37 annotation. Symbols corresponding to known synonyms or legacy names were replaced with their current HGNC/NCBI symbols (e.g., *CNT5*→*CNTN5* and *NIBAN1*→*FAM129A*). At the *IFNG* locus, the antisense transcript *IFNG-AS1* was mapped to the protein-coding gene *IFNG* for pathway interpretation. Composite labels for intergenic regions (e.g., *NPHP3*–*ACAD11*) were split into the corresponding individual genes when both were present in the GRCh37 gene set. Entries not represented as annotated genes in GRCh37 (e.g., *MYMX*, *LINC02391*, *ANKRD26P2*, and *ME2P1*) were excluded.

The resulting non-redundant list of genes derived from genome-wide significant and suggestive loci was used as the input for the PANTHER Overrepresentation Test (PANTHER v19.0; Annotation Version Release 20240620), with the Homo sapiens genome as the reference background. Fisher’s exact test with BH-FDR correction was applied. Statistical significance was defined as FDR < 0.05, and 0.05 ≤ FDR < 0.10 was considered suggestive. Fold enrichment was defined as (list %)/(reference %). UNCLASSIFIED categories were excluded from reporting.

## 5. Conclusions

We conducted a case–control genome-wide association study (GWAS) in a multicenter Japanese cohort (192 cases/15,302 controls) and identified eight genome-wide significant loci (*FHIT*, *LHX2*, *TRMT1L*, *MEGF10*, *SPATS1*, *SAMD5*, *MYT1L* and *ID4*) under favorable genomic control (λ_GC = 1.04). At the gene level (MAGMA), *FHIT*, *TRMT1L* and *MEGF10*, together with *RNF2*, *SWT1*, *VAMP1*, *TAPBPL* and *C9orf3*, achieved an FDR < 0.05. Although the pathway analysis (PANTHER) did not reach FDR significance, nominal enrichments were observed in categories including inflammation mediated by chemokine and cytokine signaling, and gonadotropin-releasing hormone receptor signaling. Taken together, these findings suggest a dual-pathway working model for iSSNHL susceptibility and clinical heterogeneity, involving immune-inflammatory mechanisms and cellular stress–homeostasis; however, pathway-level interpretations are exploratory and require replication and functional validation.

Future work should focus on replication in independent cohorts, fine mapping, and functional assays in relevant cells (e.g., expression analysis, eQTL mapping, and CRISPR perturbation) to identify causal variants and molecular mechanisms. From a clinical perspective, combining genotype with acute phase biomarkers (IFN-γ/TNFα, NF-κB target gene expression, etc.) could facilitate patient stratification to predict steroid responsiveness and optimize selective anti-inflammatory therapies.

## Figures and Tables

**Table 1:** Genome-wide significant loci associated with idiopathic sudden sensorineural hearing loss (GRCh37/hg19).

Chr	Position (hg19)	rsID	Nearest Gene	Note	Alleles (REF > ALT)	OR (95% CI)	−log10(*p*)
3	59,739,919	rs6803403	*FHIT*	overlap	G > A	0.11 (0.08–0.16)	31.997
9	126,854,522	rs72759216	*LHX2*	intergenic	C > T	3.52 (2.77–4.46)	24.595
1	185,114,837	rs6684586	*TRMT1L*	overlap	G > A	0.20 (0.14–0.28)	19.815
5	126,655,749	rs246887	*MEGF10*	overlap	C > T	0.12 (0.08–0.19)	18.787
6	44,305,662	rs184005082	*SPATS1*	intergenic	G > T	17.31 (7.12–42.11)	9.487
6	147,891,025	rs79150545	*SAMD5*	overlap	A > T	5.91 (3.23–10.80)	8.12
2	2,736,583	rs537646588	*MYT1L*	intergenic	A > T	91.45 (19.59–426.80)	8.038
6	19,931,281	rs80344881	*ID4*	intergenic	T > C	7.46 (3.69–15.07)	7.653

Results were obtained from dosage-based logistic regression adjusted for sex and PC1–PC10 (192 cases and 15,302 controls; N = 15,494). ORs (95% CIs) are reported per effect allele. “Overlap” indicates that the lead SNP lies within the gene body, whereas “Intergenic” indicates that it falls outside the annotated gene bodies; nearest genes were defined using NCBI37.3.gene.loc. Abbreviations: CI, confidence interval; OR, odds ratio; PC, principal component; GWAS, genome-wide association study.

**Table 2:** Suggestive loci from the SNP-level GWAS (5 × 10^−8^ ≤ *p* < 1 × 10^−6^).

Chr	Position (hg19)	rsID	Nearest Gene(s)	Note	Alleles (REF > ALT)	OR (95% CI)	−log10(*p*)
6	154,692,510	rs574517306	*AL357075.5*	intergenic	AT > A	12.47 (4.95–31.39)	7.065
4	35,869,706	rs78719745	*ARAP2*	intergenic	A > C	10.17 (4.35–23.79)	7.06
8	133,264,431	rs190546757	*KCNQ3*	overlap	G > A	26.17 (7.86–87.10)	6.987
12	92,701,875	rs191797614	*LINC02391*	intergenic	G > A	10.27 (4.34–24.31)	6.932
6	915,623	rs139768353	*AL356130.1*	intergenic	T > C	36.64 (9.57–140.29)	6.834
13	39,512,691	rs78023951	*ANKRD26P2*	intergenic	A > G	35.59 (9.39–134.88)	6.83
9	29,879,336	rs374180147	*ME2P1*	intergenic	C > T	76.86 (15.06–392.40)	6.748
11	100,003,980	rs570999833	*CNTN5*	overlap	C > T	26.46 (7.69–90.98)	6.696
10	43,319,165	rs181558197	*BMS1*	overlap	G > A	27.43 (7.87–95.62)	6.696
10	72,690,509	rs141094288	*AC073176.2, AC073176.1*	intergenic	A > T	13.28 (5.00–35.26)	6.684
7	45,065,153	rs532757028	*CCM2*	overlap	A > G	22.27 (6.77–73.21)	6.491
12	118,258,848	rs553208041	*KSR2*	overlap	A > G	23.90 (7.06–80.93)	6.467
13	39,806,094	rs186662456	*LHFPL6*	intergenic	G > A	21.18 (6.51–68.94)	6.4
3	132,403,996	rs147716413	*NPHP3-ACAD11, NPHP3*	overlap	T > C	16.98 (5.62–51.27)	6.291
2	169,953,552	rs146954815	*DHRS9*	intergenic	C > T	16.77 (5.55–50.68)	6.237
11	111,741,632	rs139237147	*ALG9, AP001781.2*	overlap	T > C	9.95 (4.02–24.62)	6.169
12	26,865,523	rs142776659	*ITPR2*	overlap	G > A	10.49 (4.15–26.54)	6.161
12	68,586,586	rs140008019	*IFNG-AS1*	intergenic	A > G	5.77 (2.88–11.55)	6.132
13	103,831,341	rs200995605	*SLC10A2*	intergenic	G > A	14.01 (4.92–39.86)	6.126
7	151,303,975	rs75786794	*PRKAG2*	overlap	A > G	18.69 (5.82–60.02)	6.059
7	105,262,898	rs190158134	*ATXN7L1*	overlap	G > A	10.62 (4.14–27.25)	6.045

Results were obtained from dosage-based logistic regression adjusted for sex and PC1–PC10 (N = 15,494); −log10(*p*) values are reported. ORs (95% CIs) are reported per effect allele (A1); alleles/positions are reported on the GRCh37/hg19 forward strand. Abbreviations: A1, effect allele; CI, confidence interval; OR, odds ratio; PC, principal component.

**Table 3:** Summary of conditional and joint analysis (COJO) at genome-wide significant loci.

Locus (Tag)	No. of Selected Leads (Used for Conditioning)	Post Model(s) Used	Min pC (Post-All) [chr:pos]	Call
*FHIT* (chr3)	3	excl (excluding target lead) + post-all (all selected leads)	1.09008 × 10^−4^ [3:59740419]	Residual signal remains
*TRMT1L* (chr1)	3	excl + post-all	5.12896 × 10^−8^ [1:184983740]	Residual signal remains
*LHX2* (chr9)	2	excl + post-all	2.78530 × 10^−5^ [9:126891034]	Residual signal remains
*MEGF10* (chr5)	1	post-all	5.72239 × 10^−6^ [5:125618842]	Removed by conditioning
*ID4* (chr6)	1	post-all	7.15933 × 10^−6^ [6:20068419]	Removed by conditioning
*SPATS1* (chr6)	1	post-all	5.13438 × 10^−7^ [6:44291201]	Removed by conditioning
*SAMD5* (chr6)	0	—	—	No independent SNP selected by COJO
*MYT1L* (chr2)	0	—	—	No independent SNP selected by COJO

COJO was performed with GCTA-COJO on ±2 Mb windows using the 1000 Genomes Phase 3 EAS reference for LD and GRCh37/hg19 coordinates. “No. of selected leads” indicates the number of independent SNPs selected by COJO-SLCT and used for conditioning. In the “excl” models, conditioning is undertaken on the other selected leads while excluding the target lead (for visualization); in the “post-all” models, conditioning is undertaken on all selected leads. “Min pC (post-all)” denotes the smallest conditional *p* value within the locus under the post-all model. Abbreviations: COJO, conditional and joint analysis; LD, linkage disequilibrium; EAS, East Asian; pC, conditional *p* value.

**Table 4:** Gene-based association results from MAGMA.

Chr (GRCh37)	Gene	*p*_MULTI	FDR_BH	−log10(*p*)
3	*FHIT*	2.27 × 10^−30^	3.64 × 10^−26^	29.64
1	*TRMT1L*	7.16 × 10^−16^	4.14 × 10^−12^	15.15
5	*MEGF10*	7.75 × 10^−16^	4.14 × 10^−12^	15.11
1	*RNF2*	1.55 × 10^−9^	6.21 × 10^−6^	8.81
1	*SWT1*	4.03 × 10^−8^	1.29 × 10^−4^	7.39
12	*VAMP1*	1.31 × 10^−5^	3.14 × 10^−2^	4.88
12	*TAPBPL*	1.47 × 10^−5^	3.14 × 10^−2^	4.83
9	*C9orf3*	1.57 × 10^−5^	3.14 × 10^−2^	4.81

Gene-level tests were performed with MAGMA v1.10 (SNP-wise multi model) on 16,025 autosomal genes (NCBI37.3; GRCh37/hg19), using the 1000 Genomes Phase 3 EAS as the LD reference and N = 15,494 as the sample-size modifier. Genes with Benjamini–Hochberg FDR_BH < 0.05 are listed and ordered by *p*_MULTI. Abbreviations: *p*_MULTI, MAGMA gene-based *p* value (multi model); FDR_BH, Benjamini–Hochberg false discovery rate; HGNC, HUGO Gene Nomenclature Committee; LD, linkage disequilibrium.

**Table 5:** PANTHER pathway overrepresentation analysis for the 44-gene GWAS-derived gene set.

PANTHER Pathways	Homo Sapiens— Reference List (20,580)	Number of Candidate Genes in the Present Study	Fold Enrichment	Raw *p*-Value	FDR
Interferon-gamma signaling pathway (P00035)	30	1	15.24	6.36 × 10^−2^	1.0
Angiotensin II-stimulated signaling through G proteins and beta-arrestin (P05911)	37	1	12.36	7.79 × 10^−2^	1.0
Histamine H1 receptor mediated signaling pathway (P04385)	45	1	10.16	9.39 × 10^−2^	1.0
Toll receptor signaling pathway (P00054)	57	1	8.02	1.17 × 10^−1^	1.0
Muscarinic acetylcholine receptor 1 and 3 signaling pathway (P00042)	60	1	7.62	1.23 × 10^−1^	1.0
B cell activation (P00010)	69	1	6.63	1.40 × 10^−1^	1.0
Gonadotropin-releasing hormone receptor pathway (P06664)	231	3	5.94	1.40 × 10^−2^	1.0
Endothelin signaling pathway (P00019)	87	1	5.26	1.74 × 10^−1^	1.0
Inflammation mediated by chemokine and cytokine signaling pathway (P00031)	262	3	5.24	1.95 × 10^−2^	1.0
Heterotrimeric G-protein signaling pathway-Gq alpha and Go alpha mediated pathway (P00027)	123	1	3.72	2.37 × 10^−1^	1.0
EGF receptor signaling pathway (P00018)	134	1	3.41	2.55 × 10^−1^	1.0
PDGF signaling pathway (P00047)	144	1	3.18	2.71 × 10^−1^	1.0
Huntington disease (P00029)	146	1	3.13	2.74 × 10^−1^	1.0
Wnt signaling pathway (P00057)	306	1	1.49	4.91 × 10^−1^	1.0

Overrepresentation testing was conducted with PANTHER using Fisher’s exact test with Benjamini–Hochberg FDR correction. Pathways are ordered by fold enrichment; UNCLASSIFIED categories are omitted. Abbreviations: FDR, false discovery rate; GWAS, genome-wide association study.

## Data Availability

Case cohort (iSSNHL) data. The Individual-level genomic and clinical data generated in this study contain sensitive human information and are not publicly available due to privacy and ethical restrictions under the approved protocol (IRB No. 5528). Deidentified data may be made available for research purposes upon reasonable request to the corresponding author and following approval by the relevant ethics committee(s). Control cohort data (BBJ/NBDC-JGA). Clinical data for the controls were provided by BBJ under a proposal approved by the review committee (BBJ Review Committee approval: P0233). Genotype data for the controls were provided by the National Bioscience Database Center (NBDC), Japan Science and Technology Agency, via the Japanese Genotype-phenotype Archive (JGA) under Data Access Committee–approved data-use agreements (Application ID: JDU000716002). Interested researchers can apply directly to BBJ and NBDC/JGA via their standard access procedures to obtain similar datasets. Summary-level results and code. All software and key parameters are detailed in the Materials and Methods section (PLINK v2.0 (the generalized linear model module), GCTA-COJO (options: --cojo-slct/--cojo-cond), MAGMA (multi-gene model), Eagle v2.4, and Minimac4). Upon reasonable request, we will share analysis scripts (e.g., ID harmonization rules, COJO region definitions) and aggregated summary level outputs underlying the main figures and tables, to the extent permitted by the consent and agreements.

## Supplementary Materials

The following supporting information can be downloaded at: https://www.mdpi.com/article/10.3390/ijms27041836/s1.

## Abbreviations

The following abbreviations are used in this manuscript:

BBJ	BioBank Japan
BH-FDR	Benjamini–Hochberg false discovery rate
CI	confidence interval
COJO	conditional and joint analysis
DS	dosage
EAS	East Asian
FDR	false discovery rate
GWAS	genome-wide association study
HWE	Hardy–Weinberg equilibrium
IBD	identity by descent
iSSNHL	idiopathic sudden sensorineural hearing loss
LD	linkage disequilibrium
MAF	minor allele frequency
OR	odds ratio
PC	principal component
QC	quality control
Rsq	imputation quality (Minimac Rsq)
SNP	single-nucleotide polymorphism
SSNHL	sudden sensorineural hearing loss
VCF	variant call format
IQR	interquartile range
SD	standard deviation
